# Fibropapillomatosis: A Review of the Disease with Attention to the Situation Northern Coast of Brazil

**DOI:** 10.3390/ani14121809

**Published:** 2024-06-17

**Authors:** Karoline Petrini Pinheiro da Cruz, Marco Aurélio Gattamorta, Eliana Reiko Matushima, Felipe Masiero Salvarani

**Affiliations:** 1 Instituto de Medicina Veterinária, Universidade Federal do Pará, Castanhal 68740970, PA, Brazil; karolinepetrini@gmail.com; 2 Aquário Municipal de Santos, Santos 11030600, SP, Brazil; marcogattamorta@santos.sp.gov.br; 3 Faculdade de Medicina Veterinária e Zootecnia, Universidade de São Paulo, São Paulo 05508220, SP, Brazil; ermatush@usp.br

**Keywords:** chelonian, tumor, fibropapilloma, herpesvirus, ChHV5

## Abstract

**Simple Summary:**

Fibropapillomatosis (FP) is a disease that affects sea turtles, causing the growth of benign tumors known as fibropapillomas. These tumors can develop on the skin, eyes, mouth, and internal organs of affected turtles, impacting their ability to swim, feed, and avoid predators. The primary cause of FP is believed to be a virus called Chelonid Alphaherpesvirus 5 (ChHV5), although other factors such as environmental pollution and genetics may also play a role. ChHV5 is thought to be transmitted through direct contact between turtles, or through contaminated water and surfaces in their habitats. The disease has been reported in sea turtle populations around the world, with varying prevalence rates in different regions. FP poses a significant threat to sea turtle populations, as it can reduce their overall health and reproductive success. Efforts to manage and prevent FP include monitoring sea turtle populations for signs of the disease, studying the virus and its transmission pathways, and implementing conservation measures to protect sea turtle habitats. Understanding the complex relationship between ChHV5 and FP is crucial for developing effective strategies to mitigate its impact on sea turtle populations.

**Abstract:**

Fibropapillomatosis in sea turtles is a potentially debilitating and fatal disease for which there is still a lack of knowledge, especially for specific regions of Brazil. The diagnosis is made through the observation of clinical manifestations, and despite its association with Chelonid Alphaherpesvirus 5 (ChHV5) as the etiological agent, the expression of the disease may also be related to immunological and environmental factors caused by anthropic degradation of the environment. Thus, this review aims to elucidate what is known about this disease globally, and especially in various regions of Brazil, promoting a better understanding of its evolution, spatiotemporal prevalence, and relationship with human activities. Furthermore, the review explores the molecular biology of ChHV5, including its genomic structure, replication cycle, and mechanisms of pathogenesis. The role of environmental factors, such as temperature and pollution, in modulating ChHV5 infection and FP development is also discussed. Additionally, the review summarizes current diagnostic methods for detecting ChHV5 infection in sea turtles, highlighting the importance of early detection and monitoring for effective disease management and conservation efforts. Finally, the review outlines future research directions aimed at improving our understanding of ChHV5 and developing strategies for FP control and prevention in sea turtle populations.

## 1. Introduction

Sea turtles are highly important in marine ecosystems as they contribute to the health and maintenance of coral reefs, estuaries, rocky shores and sandy beaches and are widely distributed throughout tropical and subtropical regions [1]. Of the seven species known worldwide, five occur on the Brazilian coast, namely the loggerhead turtle (*Caretta caretta*), the hawksbill turtle (*Eretmochelys imbricata*), the green turtle (*Chelonia mydas*), the olive turtle (*Lepidochelys olivacea*) and the leatherback turtle (*Dermochelys coriacea*).

They are found in different regions of Brazil, including their feeding, growth, resting, reproduction, and spawning areas. The population stocks, conservation status, and biological and behavioral conditions vary among the species and are related to factors such as predatory fishing, contamination and pollution of the seas, and maintenance of the availability and quality of breeding and feeding areas [2,3].

According to ordinance nº 148 [4] published on behalf of the Brazil Ministério do Meio Ambiente (MMA), regarding the update of the national list of extinction risk classification of species, although it includes the green turtle (*C. mydas*) in the near threatened (NT) category, some other species remain threatened with extinction on the Brazilian coast, such as the hawksbill turtle (*E. imbricata*) in the endangered (EN) category, loggerhead turtle (*C. caretta*) in the vulnerable (VU) category, leatherback turtle (*D. coriacea*) in the critically endangered (CR) category, and the olive turtle (*L. olivacea*) in the VU category. The Red List ranking of the International Union for Conservation of Nature (IUCN), Marine Turtle Specialist Group (MTSG), provides different but still worrying information for the conservation of sea turtle biodiversity on the Brazilian coast, as it includes the green turtle (*C. mydas*) in the least concern (LT) category, loggerhead (*C. caretta*) too in the LT category, leatherback (*D. coriacea*) in the CR category, hawksbill turtle (*E. imbricata*) too in the CR category, and the olive turtle (*L. olivacea*) in the VU category, all in the South Atlantic ocean subpopulation [3]. This finding justifies the maintenance of conservation and monitoring programs for all species of sea turtles that occur off the Brazilian coast [3,4,5]. There are several factors that pose risks to sea turtles, such as incidental fishing, the ingestion of solid waste, interactions with fishing gear or boats, parasitic diseases, and fibropapillomatosis (FP), which is a debilitating disease of multifactorial nature that can be fatal [6,7,8,9,10]. Even with the efforts of the TAMAR-ICMBio Project and several other entities that continue to work and provide information on these animals along the Brazilian coast, there are still gaps in knowledge about the occurrence and health status of these species in some coastal regions, particularly in the northern region of the country [11].

Hamede et al. [12] emphasized the crucial need to enhance disease surveillance and data collection to address the alarming rise in the number of wildlife illnesses linked to human activities and rapid environmental changes. Specifically in the case of FP, challenges in conducting aquatic research and acquiring high-quality relevant data have hindered the effectiveness of surveillance and information gathering efforts. When discussing research on the prevalence of FP in sea turtles, it is crucial to emphasize the importance of implementing efficient study methods to obtain data that accurately reflect the true situation. Previous research has shown that the approach taken in data collection can significantly impact data interpretation [13].

These gaps in knowledge make it difficult to determine the proportion of turtles with FP, given that sea turtles are migratory animals that circulate along the entire Brazilian coast and even in other regions of the world [11]. Thus, this review aims to contribute to the understanding of various aspects of FP in sea turtles in the context of Brazil, from its characterization to its significance for the conservation of the species, since these animals play a fundamental role in the environment, and FP may present a risk to affected individuals. Without a solid information base, it becomes increasingly difficult to gather the necessary resources to establish programs and initiatives to reduce the impact on turtles and mitigate environmental factors that contribute to FP development.

## 2. Research Findings on Knowledge of Sea Turtles and FP on the Northern Coast of Brazil

Since 1980, the breeding areas of marine species have been systematically identified in Brazil, with the active participation of the TAMAR-ICMBio Project in the main locations identified. In recent years, various institutions have conducted research and monitoring to increase our knowledge about other breeding beaches. However, there are still areas in Northern Brazil that lack sufficient information for a complete understanding [11,12].

In the state of Pará, there is a lack of research on marine turtles, particularly on the incidence of FP in these animals. This highlights the urgent need for more information to assist in the preservation of these invaluable species for the ecosystem. Especially in an area with such great potential, where the animals of the five marine turtle species found in Brazil are known to be incidentally captured in fishing gear such as gillnets, longlines, fish traps [11,14,15,16].

The study conducted by Martini et al. [12] revealed a stark lack of interest in obtaining permits from the Biodiversity Authorization and Information System (SISBIO) to study marine turtles in the northern region of Brazil, in comparison to other regions of the country. Furthermore, a significant number of research projects initiated in the region have failed to produce publishable results due to a myriad of challenges, such as limited financial resources to support the research and the difficulty of effectively monitoring the vast areas in the region.

The breeding season of marine turtles varies depending on the region and the species being studied. In Brazil, it is widely known that the nesting season begins in September and continues until April along the coastal beaches, while on the oceanic islands, it begins in December and the turtles remain in reproductive activity until June [17]. The nesting period in the Pará region is described as occurring between March and July [18].

In a study conducted in an extractive reserve in the municipality of Curuçá, along the coast of Pará, Brazil, the presence of green turtles, loggerhead turtles, hawksbill turtles, and olive ridley turtles was documented through incidental fishing captures. Additionally, nests with hatchlings of the species *C. mydas*, *E. imbricata*, and *L. olivacea* were monitored [18]. The latter species was also recorded in the Para region in a study by Silva [19], where a specimen was brought in for veterinary care due to a lacerative injury on its flipper, indicative of entanglement in fishing gear. These findings confirm the species’ presence in the region and its interaction with fishing activities. The presence of a leatherback sea turtle (*Dermochelys coriacea*) was not recorded, even with the adult individuals or nests being monitored. However, reports from survey participants indicate the capture of an adult female with eggs present, who unfortunately died after being caught in a beach seine fishing net. Another adult individual was also reported to have become entangled in a fishing net in the same year, 2009 [18].

The peak period for the capture of turtles in the fishing enclosures along the coast of Pará extends from February to June [18], aligning with the pattern observed in the region of Northeastern Brazil, specifically in the state of Ceará, where a higher incidence of captures in fishing enclosures was also noted during the first half of the year [20,21,22].

A retrospective study conducted at a Wildlife Screening and Rehabilitation Center (WSRC) in the state of Pará, Brazil, recorded the occurrence of 20 individuals, with *E. imbricata, C. mydas*, and *C. caretta* species being the most prevalent, predominantly consisting of juvenile specimens, followed by juvenile animals, and the only one adult. The majority of rescues were identified in the regions of Pará, Brazil (Table 1), with the presence of the *C. mydas* species indicating the intensive use of the area by this species in the state [23].

In a study conducted by Silva [23], the presence of turtles with lesions compatible with FP was observed in three juvenile specimens of *C. mydas*, found in the northeastern state of Pará, Brazil, in the cities of Curuçá, Salinópolis, and Maracanã (specifically on the island of Algodoal), respectively. The characteristics of the tumors followed the pattern described by other authors, mainly located in soft tissues, with distribution in the fin regions, around the eyes, neck, inguinal area, cervical region, axillary region, base of the tail, and cloaca.

The northern region of Brazil lacks comprehensive studies on marine turtles, particularly in relation to the incidence of FP in the population. This highlights the need for investments in monitoring programs to identify priority areas for the reproduction and feeding of these species. Understanding the health status of individuals in the region is crucial in developing effective conservation strategies. This information will guide efforts to protect and preserve these endangered species [12].

## 3. FP in Sea Turtles

**Figure 1:**
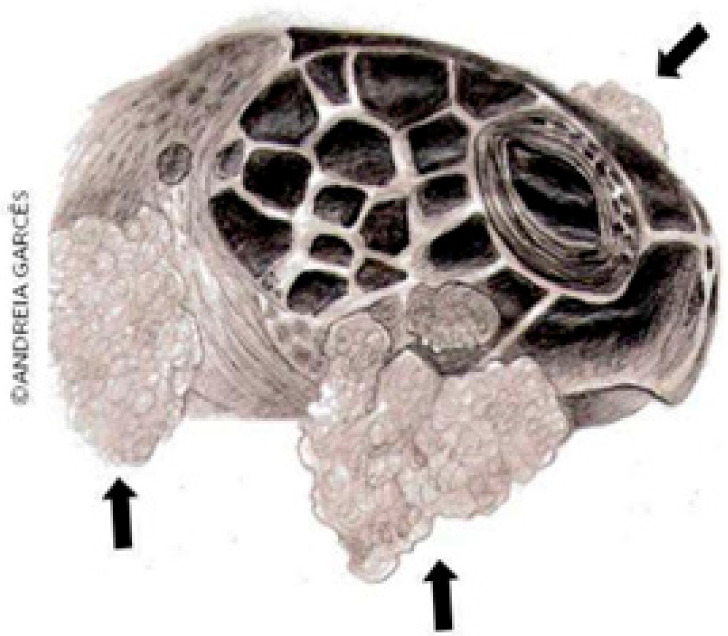
FP in a sea turtle [26].

**Figure 2:**
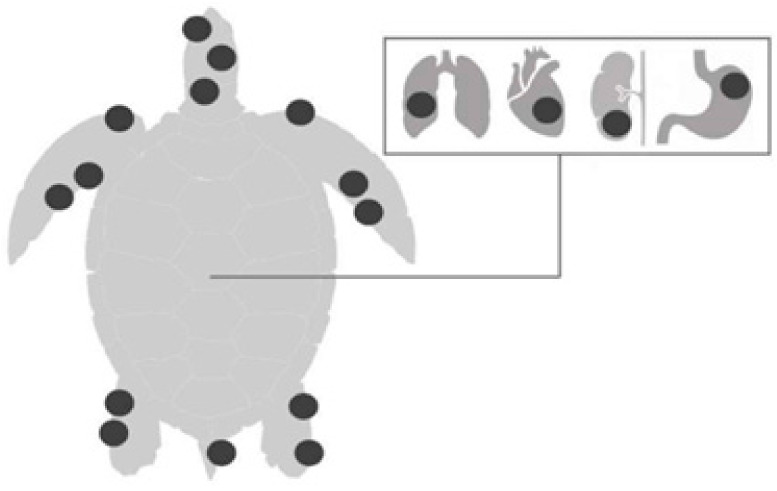
Common anatomical locations of masses on the skin (fins, neck, chin, inguinal and axillary regions, and tail) and internal organs (heart, lung, kidney, and digestive tract) of sea turtles [26].

FP is characterized by single or multiple tumors (Figure 1) that develop in various regions of the body, especially at the base of the fins and tail, and on the neck, head, and eyes; they may also occur on internal organs such as the lungs, liver, kidneys, ovaries, and heart (Figure 2). Tumors can also develop in tissues of the carapace and plastron, as well as in the cornea of affected animals [24]. The neoformations may have a verrucous, smooth or rough appearance and have different colors, depending on the pigment of the area where the tumor originated [25]. Clinical evaluations have shown that these injuries have detrimental effects on the vision of turtles, impacting their ability to search for food, evade predators, and interact with other individuals [26].

These tumors, including fibromas, cutaneous papillomas, and fibropapillomas, despite their benign course, can impair locomotion, vision, breathing, or even the apprehension of food, and depending on the stage of the disease, the animal can be weak and anemic; therefore, it is a debilitating and potentially fatal disease for sea turtles [6,7,27,28]. When associated with the presence of Chelonid Alphaherpesvirus 5 (ChHV5) as the causative agent of the disease, it is also possible to detect viral particles in oral, ocular, and cloacal secretions, blood, urine and plasma [29,30,31]. In an infected loggerhead turtle (*C. caretta*), bilateral mucoid secretion, chemosis, conjunctival hyperemia, and bilateral eyeball retraction were observed [32].

There are records of the occurrence of fibropapillomas in all species of sea turtles, and there is evidence that the green turtle (*C. mydas*) has the highest prevalence of this disease among sea turtles [33]. The curved carapace length (CCL) is a significant factor in the occurrence of the disease, given that the most frequent disease occurrence has been in turtles with CCLs greater than 30 cm, with a significant decrease in prevalence in animals with CCLs equal to or greater than 80 cm [7]. In a study conducted in Australia, it was observed that turtles affected by FP were often young animals [34]. Therefore, it is frequently observed in young animals, but it has also been diagnosed in turtles near the adult stage and less commonly in adults [35].

This difference in size between affected animals may be because younger individuals die and disappear from the locality, or they acquire immunity and mature to adults without the disease; alternatively, healthy adult individuals may have never been exposed to the potential infectious agent [33]. Sex is not a determining factor for the disease, and no significant difference in prevalence has been observed between males and females [36].

The first case of FP in Brazil occurred in 1986 in Espírito Santo, and the monitoring of FP was included in the national database of the TAMAR project which began in 2000; the presence or absence of tumors characteristic of the disease are recorded in the database. Based on this monitoring, it was possible to characterize the prevalence of the disease in different regions along the Brazilian coast, and until 2015, it was possible to observe disease-free areas, such as the oceanic islands of Atol das Rocas and Trindade [7,37,38].

Studies have sought to elucidate the relationship between the prevalence of FP and environmental quality, including heavy pollution in coastal areas, high human density, agricultural runoff and/or biotoxin-producing algae [39]. The island of Fernando de Noronha was among the areas free of FP included in TAMAR until December of 2015, when a green turtle (*C. mydas*) with a bilateral ocular nodule, characterized as a fibropapilloma, was reported. This case was associated with ChHV5, revealing the importance of constant monitoring of the disease [27].

Despite the damage caused to individuals affected by this disease, there are still conflicting discussions about whether FP is of significant importance to the survival of sea turtles. Authors such as Hamann et al. [40] have emphasized that deepening our knowledge and effectively addressing this disease is a top priority in research focused on the conservation of sea turtles. Ongoing surveillance of FP is essential for identifying changes in the spread, incidence, and severity of the disease, as stated by specialists [41]. Without a complete understanding of the disease, one cannot rule out FP as an obstacle to the survival of the species [33].

## 4. Diagnosis, Treatment, and Control of FP in Sea Turtles

The diagnosis of the disease relies on physical examination of an affected animal. Tumors are found via macroscopic visualization and can be confirmed via histopathology after the incision of a fragment of the lesion [42]. Developing sensitive and specific diagnostic tools for detecting ChHV5 in sea turtles, including rapid PCR assays and serological tests, is essential for an accurate diagnosis.

Furthermore, investigating non-invasive diagnostic methods, such as thermal imaging and biomarker detection, is crucial for the early detection of FP in sea turtles [12,33,40].

Histopathological analyses performed by Rodenbusch et al. [43] demonstrated that fibropapillomas have a papillary pattern, with the presence of melanocytes, epithelial hyperplasia, hyperkeratosis, and a nuclear halo, in addition to moderate stromal hyperplasia and dyskaryosis. In the analyses performed by Gattamorta et al. [27], fibropapilloma was characterized by hyperplastic epidermal and stromal proliferation, epithelial cells with cytoplasmic vacuolization, degeneration of epidermal cells, and fibroblast proliferation.

A significant challenge in managing FP is the absence of a specific cure or effective vaccine. Current treatment options are limited to the surgical removal of tumors as well as supportive care, which may help improve the quality of life for affected turtles but do not address the underlying cause of the disease. Research into antiviral therapies and immunomodulatory drugs holds promise for improving the treatment outcomes for FP. However, further studies are needed to evaluate the effectiveness of antiviral drugs, immunomodulatory therapies, and supportive care in treating FP and improving sea turtle health. Therefore, future scientific research involving the development of novel treatment strategies, such as the use of gene therapy or immunotherapy, with the aim of controlling tumor growth and viral replication in affected turtles is necessary [12,33,40].

To prevent and control FP, it is necessary to prevent pollution, habitat degradation, and other human activities that can weaken sea turtle immune systems and increase their susceptibility to FP [7,8,9,10]. Moreover, implementing quarantine and biosecurity measures in sea turtle rehabilitation facilities and captive breeding programs to prevent the spread of FP, along with educating the public about the importance of sea turtle conservation and the risks of FP to help reduce human activities that can contribute to the spread of the disease are prospective solutions to curb the prevalence of FP [12,33,40].

## 5. Association of FP with the ChHV5 Virus in Sea Turtles

The first studies conducted by Smith and Coates [44] did not identify viral elements in histological exams, but recent studies have suggested that FP has an infectious origin [7,28,45,46]. The most prevalent theory indicates that the virus involved in FP is ChHV5, with a taxonomic position in the *Herpesviridae* family, the Alphaherpesvirinae subfamily, and the Scutavirus genus [33,47]. Sequence analysis of fragments of the DNA polymerase region revealed the presence of ChHV5 in fibropapillomas, and several other regions of the ChHV5 genome have been studied as well [7,35,43,44,45,46,47,48,49,50,51,52].

According to Ene et al. [28], it is not clear at which stage of sea turtle development the transmission of infectious agents occurs, with the prenatal period, hatching, or time spent in the pelagic zone being possibilities. Sea turtles have a complex life history, beginning when the young are born, continuing during their migration to pelagic environments, and their subsequent return to coastal areas for foraging, and later for reproduction and nesting, making it difficult to identify the life stage and location where virus transmission occurs [53]. There is speculation about the potential spread of the virus through the dispersion and distribution of juvenile turtles to aggregation areas, driven by ocean currents. These turtles come into contact with a high density of infected marine turtles [32], possibly spreading the disease when they return to their original beach [54,55,56]. This transmission can occur through direct contact with tumors/secretions or through vectors such as marine leeches, cleaner fish, or through infected individuals and waters [53,57,58,59].

There are currently two hypotheses as follows: horizontal transmission through direct contact with tumors/secretions or through vectors and water [53], and vertical transmission as recent analyses have reported the presence of ChHV5 in hatchlings [60]. However, in another study, the hatchlings tested negative despite coming from mothers that tested positive using the same method [61].

The first studies to detect ChHV5 in the tissues of turtles with and without FP showed that amplification of the target gene sequences was rare in animals without the disease [46]. Subsequently, Quackenbush et al. [50] amplified ChHV5 in samples collected from turtles without FP, showing that the virus may be present in animals without clinical signs, i.e., the absence of clinical signs of the disease does not confirm the absence of viral infection. It is possible that active or latent forms of the virus may be more common in turtles than previously imagined and that perhaps the percentage of sick animals is lower than that of turtles infected with the virus [51]. Results from a study conducted in the coastal waters of Granada in 2020 revealed a significant finding as follows: An adult green turtle underwent necropsy due to severe emaciation and cutaneous fibropapillomas, and upon further testing, it was confirmed that several tumors tested positive for ChHV5, marking the first case of FP associated with this virus in Granada. In this region, active ChHV5 infection is considered rare [62].

Other studies have also detected ChHV5 in tissues and animals without FP, which may suggest that the presence of the virus alone is not a determinant for the development of the disease and that other factors, such as the condition of the host, the presence of the virus and environmental conditions, may be involved [7,63].

Although studies have shown the presence of the ChHV5 virus in the tumor tissues of animals with FP, this result may not be surprising, as these tumors are known to have a higher concentration of viral DNA; however, in the presence of the virus in latent infections, i.e., in animals that do not present clinical manifestations of disease, the viral DNA load in tissues tends to be lower, which represents a challenge for the detection of the presence and prevalence of the virus under these circumstances [33].

Considering this, Alfaro-Núñez et al. [64] sought to demonstrate an effective molecular testing method by studying animals that did not present clinical manifestations of the disease and had apparently healthy tissues. The authors detected the presence of ChHV5 in 100% of the samples analyzed, suggesting latent infections and corroborating the assumption that the presence of this virus in tissues, fluids, and secretions of sea turtles without tumors is indicative of the multifactorial nature of FP [51].

## 6. Multifactorial Nature of FP in Sea Turtles

Despite the strong evidence for herpesvirus involvement, other factors are likely involved in FP infection and symptom manifestation, such high nitrogen levels in watersheds that contribute to coastal eutrophication. These result in eutrophication events and the proliferation of microalgae, causing a nutritional imbalance in foraging areas, altering the immune system of turtles and thus favoring the formation of tumors, parasitism, genetic susceptibility, and biotoxins, and adverse effects from prolonged exposure to ultraviolet light, chemical pollutants, and environmental and immunological factors [7,65,66].

Water temperature may also influence the development and growth of FP lesions, with higher temperatures probably being more favorable for FP, as observed by Herbst [32] in Florida, where the change in water temperature is significant because the seasons are well defined. Studies have shown that the increased incidence of ultraviolet radiation is responsible for the onset of the disease. This is due to the ability of radiation to damage DNA and thus give rise to an increase in the mutations that cause tumors in animals. In addition, in captive turtles, tumor growth was more evident during warmer times of the year, indicating that climate change can directly impact FP [67,68,69].

In a study conducted by Garefino [70], the plasma levels of vitamin D in turtles with and without visible FP tumors were analyzed. Vitamin D plays a crucial role in immune function and overall health, and the study results indicated that prolonged exposure to sunlight significantly affected the levels of this vitamin in the marine turtles brought to a rehabilitation center. Additionally, turtles with tumors were observed to have lower levels of vitamin D and calcium, and higher levels of parathyroid hormone compared to those without tumors. Turtles housed in tanks with higher exposure to ultraviolet light showed greater increases in vitamin D levels and more effective recovery. These findings suggest that sun exposure can benefit the health and recovery of green turtles with FP in rehabilitation centers.

Jones et al. [34] investigated the impact of water quality variables on FP that could directly or indirectly impact turtles, such as metals and pesticides with potential toxic effects on turtles, as well as dissolved inorganic nitrogen and total suspended solids that could harm seagrass growth, a vital food source for green turtles [71]. These could indirectly affect the health and presence of green turtles in their feeding areas. However, the study yielded data that, while relevant, did not show significant importance in indicating a relationship between FP and the water quality parameters analyzed. Overall, the research finding of Jones et al. [34] shed light on the potential impacts of certain water quality variables on turtle health, but further investigation is needed to determine the exact relationship between FP and water quality factors.

According to James’ data analysis [62], it is clear that the genetics of ChHV5 does not appear to be a determining factor in the evolution or variation of the disease. Over two decades of sampling, it was observed that the virus strains remained constant and highly similar. Therefore, it is likely that environmental or host processes play a key role in the development and progression of the disease.

Sea turtles are considered sentinel species, representing the health status of marine ecosystems [39]. Molecular studies [45,50,57,72] have demonstrated the presence of ChHV5 in animals with and without FP, and due to the possible relationship between the development of FP and the quality of the environment where the turtles live, and because the disease may be stimulated by environmental factors, it is extremely important to understand both the prevalence of the disease in these species and the quality of the water where these animals live so that an efficient management plan can be developed to support the maintenance and conservation of the environment and sea turtles [25,73,74].

Therefore, monitoring FP is crucial for understanding its epidemiology, distribution patterns, and severity [27,74]. As proposed by Jones et al. [33], future studies should also take environmental factors into account, such as water quality, and evaluate not only the presence of FP but also possible latent infections without apparent clinical signs. Another important aspect of FP research is understanding its epidemiology and transmission dynamics. Factors such as water temperature, pollution, and habitat degradation may influence the prevalence and severity of FP outbreaks. By identifying these factors, conservation efforts can be targeted towards reducing the impact of these environmental stressors on sea turtle populations. Future research on FP should also focus on improving diagnostic tools for early detection of the disease. Rapid and accurate diagnosis is crucial for implementing timely treatment and control measures. Advances in molecular biology and imaging techniques may help improve diagnostic accuracy and efficiency. In conclusion, FP remains a significant threat to sea turtle populations worldwide, highlighting the need for continued research and conservation efforts. By improving our understanding of the disease and developing effective management strategies, we can help protect these iconic marine reptiles for future generations.

## 7. Conclusions

Research on FP in sea turtles is crucial for understanding its current state, and ongoing developments are vital to the conservation of these species and the environment in which they live, since there are strong relationships between these elements, in addition to the presence of ChHV5. This study contributes to a deeper understanding of FP, highlights the need for further research, and reveals that the search for more information about the interaction of animals with viruses, transmission modes, and the mechanisms that lead to the development of the disease should be prioritized to support the establishment of management methods and species conservation. Further research is needed in areas with limited data on sea turtle populations and FP incidence, such as in the northern region of Brazil. As these cosmopolitan creatures inhabit various locations, their conservation, especially in breeding grounds, is essential for a sustainable future of the sea turtle.

## Figures and Tables

**Table 1:** Frequency of occurrence of sea turtles in the mesoregions of Pará, Brazil.

Mesoregions	City	Individuals per Species			Total
		*C. mydas*	*E. imbricata*	*C. caretta*	
Northeast of Pará	Maracanã (Algodoal island)	1	0	0	1
	Bragança	0	0	1	1
	Curuçá	2	0	0	2
	Salinópolis	1	10	0	11
Marajó Archipelago	Salvaterra	1	0	0	1
	Santa Cruz do Arari	0	0	1	1
	Ponta de pedra	1	0	0	1
Undefined		2	0	0	2

Source: Adapted from Silva, 2023 [23].

## Data Availability

Not applicable.
